# Induction and Aggravation of the Endoplasmic-Reticulum Stress by Membrane-Lipid Metabolic Intermediate Phosphatidyl-*N*-Monomethylethanolamine

**DOI:** 10.3389/fcell.2021.743018

**Published:** 2022-01-06

**Authors:** Yuki Ishiwata-Kimata, Quynh Giang Le, Yukio Kimata

**Affiliations:** Division of Biological Science, Graduate School of Science and Technology, Nara Institute of Science and Technology, Ikoma, Japan

**Keywords:** unfolded protein response, yeast, phospholipids, endoplasmic reticulum, membrane

## Abstract

Phosphatidylcholine (PC) is produced via two distinct pathways in both hepatocytes and yeast, *Saccharomyces cerevisiae*. One of these pathways involves the sequential methylation of phosphatidylethanolamine (PE). In yeast cells, the methyltransferase, Cho2, converts PE to phosphatidylmonomethylethanolamine (PMME), which is further modified to PC by another methyltransferase, Opi3. On the other hand, free choline is utilized for PC production via the Kennedy pathway. The blockage of PC production is well known to cause endoplasmic reticulum (ER) stress and activate the ER-stress sensor, Ire1, to induce unfolded protein response (UPR). Here, we demonstrate that even when free choline is sufficiently supplied, the *opi3Δ* mutation, but not the *cho2 Δ* mutation, induces the UPR. The UPR was also found to be induced by *CHO2* overexpression. Further, monomethylethanolamine, which is converted to PMME probably through the Kennedy pathway, caused or potentiated ER stress in both mammalian and yeast cells. We thus deduce that PMME *per se* is an ER-stressing molecule. Interestingly, spontaneously accumulated PMME seemed to aggravate ER stress in yeast cells. Collectively, our findings demonstrate the multiple detrimental effects of the low-abundance phospholipid species, PMME.

## Introduction

The endoplasmic reticulum (ER) is a membrane-bound cellular compartment that facilitates the folding of secretory, cell surface, and organelle proteins. Moreover, membrane lipids are mainly synthesized on the ER. Impaired ER performance, namely ER stress, dynamically changes the transcriptome of eukaryotic cells. This cytoprotective response is called the unfolded protein response (UPR) or the ER stress response, and is at least partly governed by the ER-located transmembrane endoribonuclease, Ire1 (Walter and Ron, 2011). In budding yeast *Saccharomyces cerevisiae* (hereafter referred to as yeast) cells, Ire1 self-associates and promotes splicing of the *HAC1* mRNA, the mature form of which is translated into a nuclear transcription factor protein (Hac1) upon ER stress. Hac1 induces a large number of UPR target genes, which include those encoding ER-located molecular chaperones and lipid biosynthesis enzymes (Travers et al., 2000; Kimata et al., 2006). ER stress is frequently accompanied by ER accumulation of unfolded client proteins, which is directly sensed by Ire1 (Kimata et al., 2007; Gardner and Walter, 2011; Kimata and Kohno, 2011; Le and Kimata, 2021).

On the other hand, certain stress stimuli that disturb membrane lipid homeostasis, namely lipid-bilayer stress (LBS), is thought to be another type of ER stress that activates Ire1 independently of the impairment of ER protein folding in yeast and animal cells (Promlek et al., 2011; Volmer et al., 2013; Hou et al., 2014). Saturation of the phospholipid acyl tails is a well-documented example of LBS and is directly sensed by Ire1 (Pineau et al., 2009; Volmer et al., 2013). Halbleib et al. (2017) proposed that the transmembrane domain of Ire1 carries an amphipathic helix segment that promotes self-association of Ire1 upon a change in lipid bilayer properties.

The composition of phospholipid hydrophilic heads can also affect the ER stress status. Phosphatidylcholine (PC) is a major component of biological membranes and is partly produced from another phospholipid, phosphatidylethanolamine (PE) via sequential methylation reactions (Lagace and Ridgway, 2013). Phosphatidylmonomethylethanolamine (PMME) is an intermediate of this reaction, which is carried out on the ER membrane. Previous studies reported UPR induction upon impairment of PC synthesis in yeast and animal cells, suggesting that an imbalanced PE/PC ratio causes ER stress (van der Sanden et al., 2003; Thibault et al., 2012; Gao et al., 2015). Nevertheless, our present study demonstrates that ER stress is induced not only by PC shortage, but also by the accumulation of PMME. Further, we propose that endogenously and spontaneously accumulated PMME is likely to aggravate ER stress in yeast cells.

## Materials and Methods

### Yeast Culture

Two standard media, YPD medium (1% yeast extract (Bacto), 2% peptone (Bacto), and 2% glucose) and synthetic dextrose (SD) medium (Mai et al., 2018) were used to culture yeast cells. Unless otherwise noted, yeast cells were grown aerobically and exponentially at 30°C in liquid media. After the addition of chemicals to the culture media, the cells were further incubated under the same conditions. To support the growth of *opi3Δ* and/or *cho3Δ* strains, SD medium was supplemented with choline (1 mM final). For the agar plates, SD medium was soldified with 2% Bacto agar. Uracil/5-fluoroorotic acid (5-FOA) SD agar plates contained 100 mg/L uracil and 1.0 g/L 5-FOA.

Culture optical density was monitored using the spectrophotometer, BioRad Smartspec 3000.

### Yeast Strains and Plasmids

Plasmids pRS316-IRE1 (the *URA3* selectable marker; Promlek et al., 2011) and pRS313-IRE1 (the *HIS3* selectable marker; Kimata et al., 2004) are yeast YCp-type single-copy plasmids that carry the *IRE1* gene (open reading frame plus the 5′- and 3′-untranslated regions). Plasmid pYT-TDH3p-PMA1-mCherry (Mai et al., 2019) is also a yeast YCp-type single-copy plasmid carrying the *URA3* selectable marker. Herein, the *PMA1*-coding sequence on pYT-TDH3p-PMA1-mCherry was replaced by a *CHO2*-coding sequence to generate pYT-TDH3p-CHO2-mCherry, which was used for expression of mCherry-tagged Cho2 under the control of the constitutive strong promoter of *TDH3*. Plasmid pRS316 (Sikorski and Hieter, 1989) was used as a control empty vector. Plasmid pPM28 was used to express eroGFP (Merksamer et al., 2008).

Unless otherwise noted, we used the standard yeast strain BY4741 (*MAT* ***a*** *his3Δ1 leu2Δ0 met15Δ0 ura3Δ0*) as the wild-type strain. Single-gene-deletion mutants of BY4741 (*xxx::KanMX4*) were obtained from EUROSCARF (http://www.euroscarf.de/). To obtain an *opi3::HIS3MX* strain, we transformed the EUROSCARF *opi3::KanMX4* strain with the *HIS3MX* gene carried on pUG27 plasmid (Gueldener et al., 2002). The *opi3::HIS3MX* construct was PCR-amplified from the resultant transformant and used for transformation of the EUROSCARF *cho2::KanMX4* strain, which yielded a *cho2::KanMX4/opi3::HIS3MX* strain.

Another yeast strain KMY1516 (*MATα ura3 his3 trp1 ire1::TRP1 UPRE-lacZ::LYS2 UPRE-GFP::LEU2*; Kimata et al., 2004) was also employed in this study. After transformation with pRS316-IRE1, KMY1516 was further transformed with the *xxx::KanMX4* constructs and/or the *opi3::HIS3MX* construct (xxx represents *opi3* or *cho2*), which had been PCR-amplified from the EUROSCARF strains or their derivative to generate gene-deletion mutants. The resulting *ire1::TRP1*/*xxx::KanMX4* strains (or the *ire1*:*TRP1*/*cho2::KanMX4*/*opi3::HIS3MX* strain) containing pRS316-IRE1 were further transformed with pRS313-IRE1 or its mutants, and cultured on uracil/5-FOA-containing agar plates to counter-select pRS316-IRE1. The *IRE1* mutants were generated as previously described (Kimata et al., 2007; Tran et al., 2019).

### Mammalian Cell Culture

HeLa cells (0.15 × 10^5^ cells) were inoculated in six-well dishes (Corning) with 3 ml Dulbecco’s modified Eagle’s medium supplemented with 10% FCS. The dishes were incubated at 37°C (5% CO_2_) for 2 days.

### RNA Analysis

Total RNA samples, which were extracted from yeast cells using the hot-phenol method (Collart and Oliviero, 2001), were subjected to RT-PCR in which the poly(dT) RT primers and the *HAC1*-specific PCR primers were employed (Promlek et al., 2011; Mai et al., 2018). As the forward and reverse PCR primers interposed the *HAC1*-intron sequence, RT-PCR yielded different-sized products from unspliced (*HAC1u*) and spliced (HAC1i) *HAC1* mRNAs. The RT-PCR products were electrophoresed on a 2% agarose gel, and the ethidium bromide-fluorescent image was captured with a UV-transilluminating imager E-Box (Vilber Lourmat). The gel images were analyzed using ImageJ software (https://imagej.nih.gov/ij/), and the *HAC1* mRNA-splicing efficiency was calculated using the following formula:

$$\mathrm{The}\;\mathrm{HAC1}\;\mathrm{mRNA}-\mathrm{splicing}\;\mathrm{efficiency}=100\times \frac{\left(\mathrm{HAC1i}\;\mathrm{band}\;\text{intensity}\right)}{\left\{\left(\mathrm{HAC1i}\;\mathrm{band}\;\text{intensity}\right)+\left(\mathrm{HACL1u}\;\mathrm{band}\;\text{intensity}\right)\right\}}$$

To check the XBP1 mRNA splicing pattern in HeLa cells, we extracted total RNA and performed RT-PCR analysis as described by Tsuchiya et al. (2018). The sequences of the human XBP1 specific PCR primers were 5′-TTA​CGA​GAG​AAA​ACT​CAT​GGC​C-3′ and 5′-GGG​TCC​AAG​TTG​TCC​AGA​ATG​C-3′.

### Phosphatidylmonomethylethanolamine Detection

For steady-state radiolabeling, cells were grown in YPD or SD medium (supplemented with 1 mM choline) containing 370 kBq/ml ^32^P-orthophosphate (NEX053, PerkinElmer) for more than 4.5 h (2–3 generations). After centrifugal harvest, cells (approximately equivalent to OD_600__=_ 1.0) were washed once with water, were suspended in 100 µl of chloroform:methanol (1:1), and were lysed by bead-beating with 100 µl of glass beads (425–600 µm; Sigma-Aldrich). The cell lysates were mixed with 200 µl of chloroform:methanol (2:1) and clarified by centrifugation (15,000 × *g*, 1 min). The supernatants were further mixed with 50 µl of chloroform-methanol (2:1), 50 µl of chloroform, and 150 µl of water. After agitation, the mixtures were centrifuged at 15,000 × *g* for 5 min, and the organic bottom layers were dried *in vacuo* and analyzed by thin-layer chromatography (TLC) as described by Sakakibara et al. (2015). Autoradiographs of the TLC plates were captured using the phosphor imaging system Amersham Typhoon and analyzed using ImageJ software.

### PC Detection

After harvest by centrifugation, cells (equivalent to OD_600_ = 40.0) were subjected to lipid extraction as described for PMME detection. The vacuum-dried lipid samples were solubilized in PBS containing 1% Triton X-100 and enzymatically assayed for PC concentration using the colorimetric PC detection kit, LabAssay^TM^ Phospholipid (Fujifilm).

### Fatty-Acid Analysis

In accordance with the Microbial Identification (MIDI) protocol (Sasser, 2001), cellular fatty acids were converted into fatty acid methyl esters (FAMEs) through saponification and methylation. The resulting FAME samples were analyzed by gas chromatography, which was performed by TechnoSuruga Laboratory Co., Ltd. (Shizuoka, Japan).

### BiP Sedimentation Assay

As described in our previous report (Mai et al., 2018), yeast cells were disrupted by glass bead beating in a Triton X-100-containng buffer. The cell lysates were separated by ultracentrifugation (160,000 × *g* for 3 h) and analyzed by anti-BiP Western blotting.

### Fluorescence Microscopy

Cells carrying the eroGFP-expression plasmid, pPM28, were observed under SP8 FALCON (Leica) with the 100×/1.40 HC PL APO CS2 objective lens. For excitation, a 405 nm diode laser (UV/violet-light excitation, 67% output) and a 496 nm white-light laser (blue-light excitation, 100% output) were employed. For detection, a hybrid detector (gating 492–571 nm) was employed. The pinhole size was 1.70 AU. The resulting images were processed as previously described (Phuong et al., 2021).

### Statistics

The culture optical density, the *HAC1* mRNA-splicing efficiency, the Western-blot band densities, and the PMME and PC contents were determined from triplicate cultures, and were subjected to calculation of averages and standard deviations. To obtain *p-*values, two-tailed unpaired *t*-tests were performed using Microsoft Excel.

## Results

### Phosphatidylmonomethylethanolamine Causes and Aggravates Endoplasmic Reticulum Stress

**FIGURE 1:**
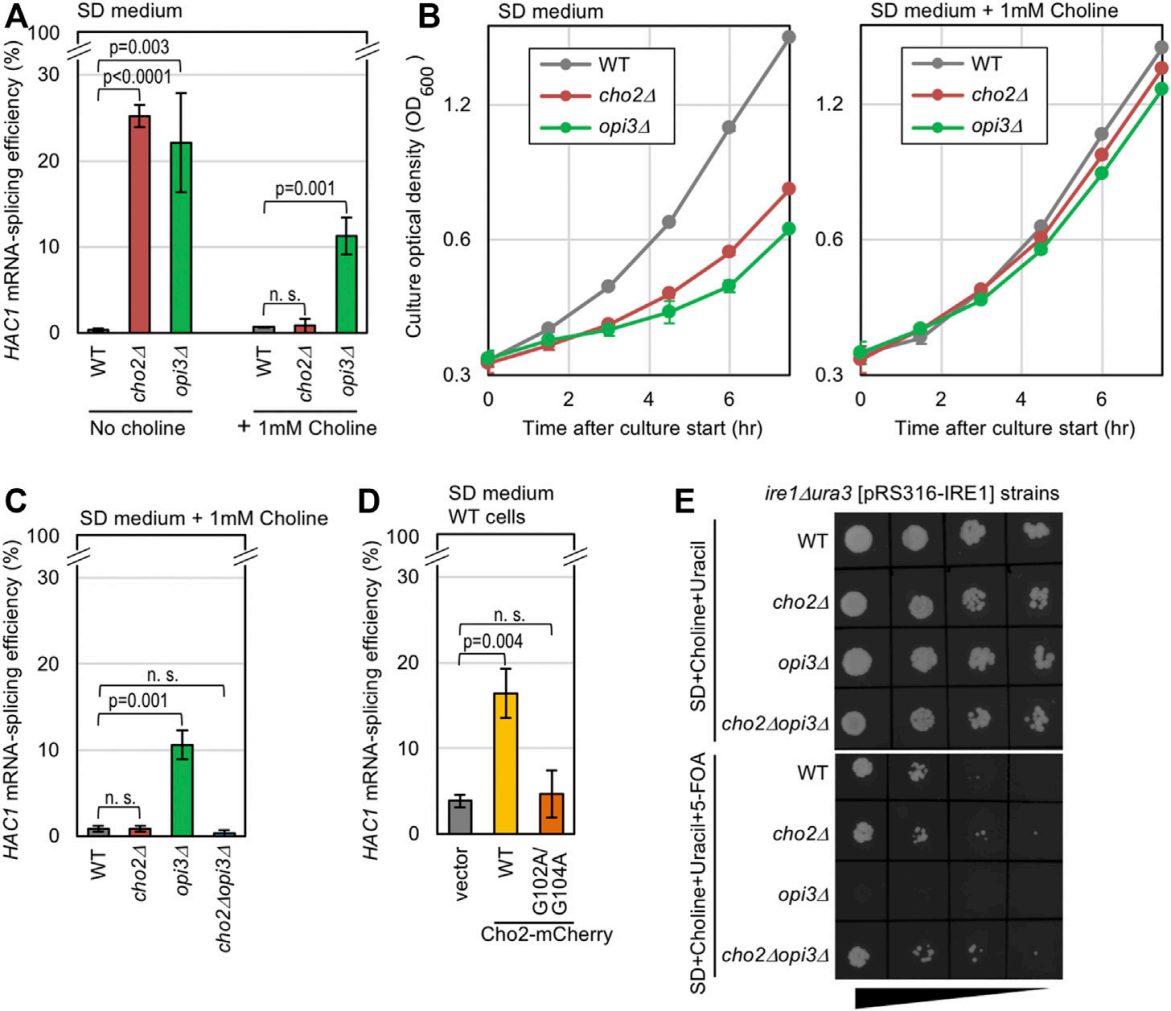
Induction of UPR by genetic manipulations that causes PMME accumulation in yeast cells. **(A–C)** Wild-type (WT) yeast strain BY4741 and its gene-deletion mutants were grown in SD medium (with or without 1 mM choline) and were checked for *HAC1*-mRNA splicing and culture density. **(D)** BY4741 cells (WT cells) transformed with the Cho2-mCherry expression plasmid pYT-TDH3p-CHO2-mCherry (WT), its G102A/G104A Cho2-mCherry variant, or the empty vector pRS316 were grown in SD medium and were checked for *HAC1*-mRNA splicing. **(E)** *ire1Δura3* yeast cells (KMY1516) carrying the *URA3*/*IRE1* plasmid pRS316-IRE1 and the indicated gene-deletion mutations were grown in SD medium containing 1 mM choline and 50 µg/ml uracil for approximately seven generations. After adjustment to OD_600_ 0.1, the resulting cultures were 5-fold serially diluted, and 5-µl aliquots were spotted onto the indicated agar plates, which were then incubated for 3 days before picturing. ns, not significant (*p* > 0.05).

In yeast cells, Cho2 acts as the PE methyltransferase for the conversion of PE to PMME, which is further converted to PC by another phospholipid methyltransferase, Opi3 (de Kroon, 2007; Supplementary Figure S1A). As previously reported (Thibault et al., 2012), Ire1 is activated to induce *HAC1-*mRNA splicing in yeast *cho2Δ* cells and *opi3Δ* cells cultured in SD medium, probably because a shortage of PC causes LBS (Figure 1A, the “No choline” condition). As PC is also produced through the Kennedy pathway, through which free choline is conjugated to diacylglycerol (Gibellini and Smith, 2010; Supplementary Figure S1A), growth retardation of *cho2Δ* cells and *opi3Δ* cells was rescued by the addition of choline into the medium (Figure 1B). Nevertheless, *opi3Δ* cells, but not *cho2Δ* cells, were found to exhibit considerable *HAC1*-mRNA splicing even in the presence of choline (Figure 1A, the “1 mM choline” condition). In the experiment shown in Supplementary Figures S1B,C, we monitored phospholipid composition through steady-state ^32^P labeling of cells and thin-layer chromatography. Consistent with a previous report by Sakakibara et al. (2015), PMME accumulation in wild-type cells and *opi3Δ* cells was completely abolished by the introduction of the *cho2Δ* mutation. Importantly, the *HAC1*-mRNA splicing in *Δopi3Δ* cells was also abolished by the introduction of the *cho2Δ* mutation (Figure 1C).

We next employed yeast cells producing Cho2 tagged with mCherry at the C-terminus (wild-type Cho2-mCherry) under the control of the strong *TDH3* promoter. While wild-type Cho2-mCherry supported the growth of *cho2Δ* cells in SD medium that did not contain choline, such support was not observed when Cho2-mCherry carried the G102A/G104A point mutation in the putative enzymatic reaction center of the Cho2 moiety (Shields et al., 2003; Supplementary Figure S2A). Therefore, as expected, the G102A/G104A mutation is likely to inactivate Cho2. Consistent with this observation, the high expression of wild-type Cho2-mCherry resulted in an increase in the cellular abundance of PMME, which was abolished by the G102A/G104A mutation (Supplementary Figure S3). Importantly, high expression of wild-type Cho2-mCherry, but not of its G102A/G104A variant, induced the *HAC1*-mRNA splicing in wild-type (*CHO2OPI3*) yeast cells (Figure 1D). Therefore, we deduce that, in addition to PC shortage, PMME accumulation causes ER stress.

The deletion of the *IRE1* gene is known to considerably worsen the growth of yeast cells upon ER stress (Cox et al., 1993). In the experiment shown Figure 1E, we employed *ura3Δire1Δ* yeast cells carrying a *URA3*/*IRE1* plasmid, which was counter-selected by 5-FOA (Boeke et al., 1984). This strain could not grow on agar plates containing 5-FOA and choline when carrying the *opi3Δ* mutation, but grew well when carrying the *cho2Δ* mutation, the *cho2Δopi3Δ* mutation, or the intact *CHO2OPI3* genes (wild-type; WT). Thus, the *ire1Δ* mutation abolished the growth of *opi3Δ* cells, but not that of wild-type, *cho2Δ*, or *cho2Δopi3Δ* cells in the presence of choline. Therefore, PMME accumulation is likely to harm cells through the induction of ER stress.

The ER stress induced by PMME accumulation is unlikely to be sufficient to cause severe growth retardation. As shown in Figure 1B, the growth rate of *opi3Δ* cells was similar to that of wild-type cells in the presence of choline. Moreover, the growth of wild-type cells was not retarded by the high expression of wild-type Cho2-mCherry (Supplementary Figure S2B).

**FIGURE 2:**
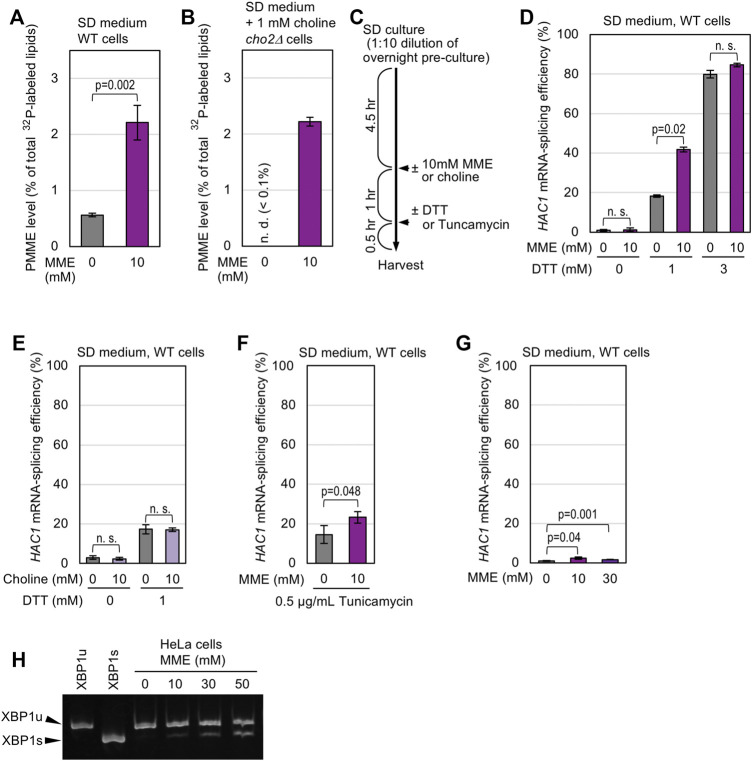
Induction or enhancement of the Ire1-mediated mRNA splicing by extracellularly supplemented MME in yeast and mammalian cells. **(A)** Wild-type (WT) yeast cells (BY4741) were grown in SD medium containing ^32^P-orthophosphate, which was supplemented with or without 10 mM MME before further culturing for 1 h. After harvesting, their lipidic extracts were assayed for PMME levels as shown in Supplementary Figure S1. **(B)** Except for the supplementation of culture medium with choline, the same analysis was performed as in **(A)** using the *cho2Δ* mutant version of BY4741. **(C)** As illustrated, cells were grown in SD medium, into which MME (or choline) and/or DTT (or tunicamycin) were added. **(D–F)** After the chemical treatment shown in **(C)**, BY4741 cells (WT cells) were checked for *HAC1*-mRNA splicing. **(G)** BY4741 cells (WT cells) were cultured in SD medium containing the indicated concentrations of MME for 1 h and were checked for *HAC1*-mRNA splicing. **(H)** After addition of PMME into the medium and further culture for 1 h, HeLa cells were examined for XBP1-mRNA splicing using RT-PCR. On the “XBP1u” and “XBP1s” lanes, the PCR products from cDNAs of unspliced (XBP1u) and spliced (XBP1s) mRNAs were loaded as band-position markers. ns, not significant (*p* > 0.05).

To further confirm our proposition that PMME *per se* causes ER stress, we added free monomethylethanolamine (MME; 10 mM) to yeast cultures, resulting in an modest increase in cellular PMME abundance (Figure 2A; Supplementary Figure S3). MME is likely to be converted to PMME via the Kennedy pathway, as a similar phenomenon was observed even when cells carried the *cho2Δ* mutation (Figure 2B). As shown in Figure 2C, we induced ER stress by adding an ER-stressing reagent, dithiothreitol (DTT) or tunicamycin, into yeast cultures containing or not containing MME or choline. In the absence of ER stressors (Figure 2D; DTT 0 mM), exogenously added MME did not induce *HAC1*-mRNA splicing in wild-type cells. This is probably because, unlike the *opi3Δ* mutation or the high expression of wild-type Cho2-mCherry, the extracellular addition of MME increased the cellular PMME abundance only moderately (Supplementary Figure S3). Moreover, 3 mM DTT led to high-level *HAC1*-mRNA splicing independently of MME (Figure 2D). Nevertheless, MME intensified the *HAC1*-mRNA splicing induced by low-dose (1 mM) DTT (Figure 2D). Unlike MME, choline did not enhance the *HAC1* mRNA-splicing level even when DTT (1 mM) was added to the cultures (Figure 2E). MME also boosted the *HAC1*-mRNA splicing that was induced by tunicamycin, although not strongly (Figure 2F).

Even when MME was added to yeast culture at a higher concentration (30 mM), *HAC1*-mRNA splicing was only marginal in the absence of another ER stress stimulus (Figure 2G). To monitor *HAC1*-mRNA splicing, we did not treated yeast cells with a higher concentration of PMME, which considerably inhibited cellular growth (Supplementary Figure S4). We then asked what occurs in the case of mammalian cells. IRE1α is the major paralog of mammalian Ire1 family proteins and promotes the splicing of XBP1 mRNA upon ER stress (Walter and Ron, 2011). MME induced the XBP1-mRNA splicing in mammalian HeLa cells in a dose-dependent manner even without other ER-stress stimuli (Figure 2H).

### Phosphatidylmonomethylethanolamine Causes Lipid-Bilayer Stress and Aggravates Dithiothreitol-Induced Proteotoxicity

Sakakibara et al. (2015) reported that the cellular accumulation of PMME caused by the *opi3Δ* mutation inhibits mitophagy in yeast cells. If PMME accumulation in *opi3Δ* cells induces ER stress and triggers UPR simply via the inhibition of mitophagy, *HAC1*-mRNA splicing may be provoked in cells carrying genetic mutations that inhibit mitophagy. However, as shown in Supplementary Figure S5, such was not observed in the case of the *atg32Δ* mutation or the *atg5Δ* mutation, which is known to abolish mitophagy (Kanki et al., 2009; Okamoto et al., 2009; Kanki et al., 2015).

**FIGURE 3:**
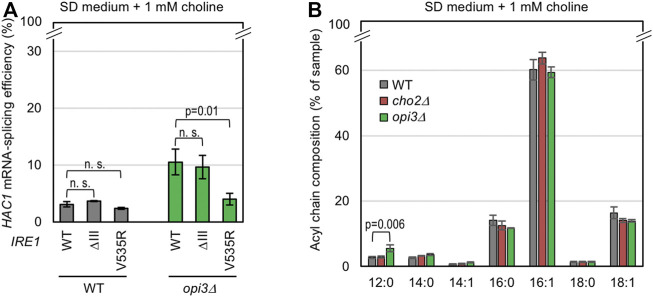
Change in the membrane-lipid composition by PMME in yeast cells. **(A)** The KMY1516 strain (*ire1Δ*) and its *opi3Δ* mutant carrying the *IRE1* plasmid pRS313-IRE1 (wild-type (WT) or the indicated mutants) were grown in SD medium containing 1 mM choline and were checked for *HAC1* mRNA splicing. **(B)** Wild-type (WT) yeast strain BY4741 and its mutants were grown in SD medium containing 1 mM choline and were checked for their lipid fatty-acid composition. ns, not significant (*p* > 0.05).

As described in the Introduction section, it is widely accepted that Ire1 directly senses ER accumulation of unfolded proteins (Kimata et al., 2007; Gardner and Walter, 2011). In the case of yeast Ire1, this ability is compromised by a luminal-domain partial deletion, namely the ΔIII mutation (Kimata et al., 2007; Promlek et al., 2011; Tran et al., 2019). On the other hand, the transmembrane domain of Ire1 serves as a sensor for LBS (Volmer et al., 2013; Halbleib et al., 2017). Halbleib et al. (2017) and Tran et al. (2019) previously indicated that this ability of Ire1 is compromised by a point mutation, V535R, which is located on the amphipathic helix of the transmembrane domain. Therefore, by using these Ire1 mutants, we can categorize stimuli that cause ER stress into two types (Tran et al., 2019). As shown in Figure 3A, the V535R mutation, but not the ΔIII mutation, of Ire1 attenuated the UPR induced by the *OPI3*-gene deletion. Thus, we deduce that high accumulation of PMME due to the *opi3Δ* mutation activates Ire1 by triggering LBS.

We then checked if the *opi3Δ* mutation affects the fatty-acid composition of lipidic molecules. In the experiment shown in Figure 3B, yeast cells were subjected to alkaline saponification, and the resulting fatty acids were methylated and quantitatively detected by gas chromatography. This revealed that the *opi3Δ* mutation, but not the *cho2Δ* mutation, increased the proportion of lauric acid (C12:0) (Figure 3B).

**FIGURE 4:**
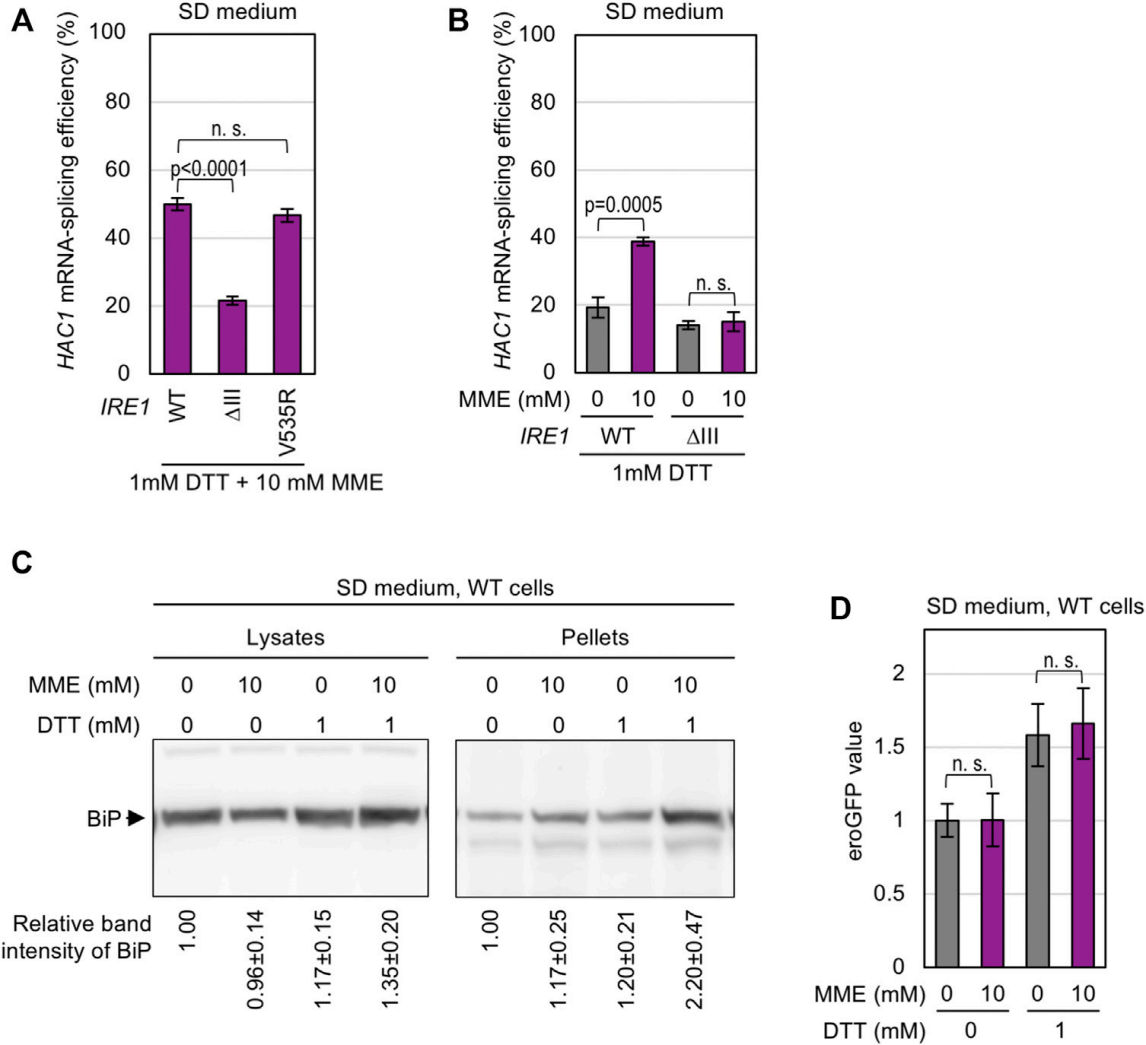
Aggravation of DTT-induced aggregation of ER proteins by PMME. **(A,B)** The KMY1516 strain (*ire1Δ*) carrying the *IRE1* plasmid pRS313-IRE1 (wild-type (WT) or the indicated mutants) was grown in SD medium and treated with 10 mM PMME and/or 1 mM DTT or not treated as shown in Figure 2C. The cells were then checked for *HAC1*-mRNA splicing. **(C)** Wild-type yeast BY4741 cells (WT cells) were grown in SD medium and treated with 10 mM PMME and/or 1 mM DTT or not treated as shown in Figure 2C. The cells were then subjected to the BiP sedimentation assay. The panels represent anti-BiP Western-blotting images of the total cell lysates (equivalent to 0.025 OD_600_ cells) and the pellet samples (equivalent to 0.25 OD_600_ cells). **(D)** BY4741 cells (WT cells) carrying the eroGFP expression plasmid pPM28 were grown in SD medium and treated with 10 mM PMME and/or 1 mM DTT or not treated as shown in Figure 2C. The cells were then observed under a fluorescence microscope. The eroGFP values are normalized against that of non-treated cells, which is set at 1.00. ns, not significant (*p* > 0.05).

On the other hand, the strong *HAC1*-mRNA splicing induced by co-treatment of cells with MME and low-dose (1 mM) DTT was compromised by the ΔIII mutation (Figure 4A). Unlike the case of wild-type Ire1, MME did not boost the low-level *HAC1*-mRNA splicing triggered by 1 mM DTT when cells carried ΔIII Ire1 (Figure 4B). Thus, we presume that this stress stimulus impairs protein folding in the ER. To support this idea, we performed BiP sedimentation analysis (Figure 4C) using a method developed in our previous studies (Promlek et al., 2011; Mai et al., 2018). Yeast cells were lysed in the presence of a mild detergent, Triton X-100, and subjected to ultracentrifugation. ER-accumulated unfolded proteins tend to form BiP-bound aggregates, the amount of which is estimated by anti-BiP Western blot analysis of the pellet fraction. As shown in Figure 4C, BiP was abundantly carried in the pellet fraction obtained from wild-type cells dually treated with low-dose DTT and MME. This result suggests that PMME aggravates DTT-induced disturbance of ER protein folding.

We next checked the disulfide bond-forming ability of the ER using the cells producing eroGFP, an ER-located GFP variant that changes its excitation spectrum depending on intracellular disulfide-bond formation. After chemical treatment, the cells were illuminated by two different wavelength laser beams, and the ratio of the two fluorescent signals was expressed as the eroGFP value. When eroGFP is reduced in the ER, cells show a higher eroGFP value (Merksamer et al., 2008). As shown in Figure 4D, treatment of cells with 1 mM DTT increased the eroGFP value, which was not further elevated by MME. Therefore, we presume that PMME does not aggravate the DTT-induced ER stress directly by stimulating the activity of DTT to reduce protein disulfide bonds.

### Spontaneously Accumulated Phosphatidylmonomethylethanolamine is Likely to Affect the Endoplasmic Reticulum Stress Status in Yeast Cells

**FIGURE 5:**
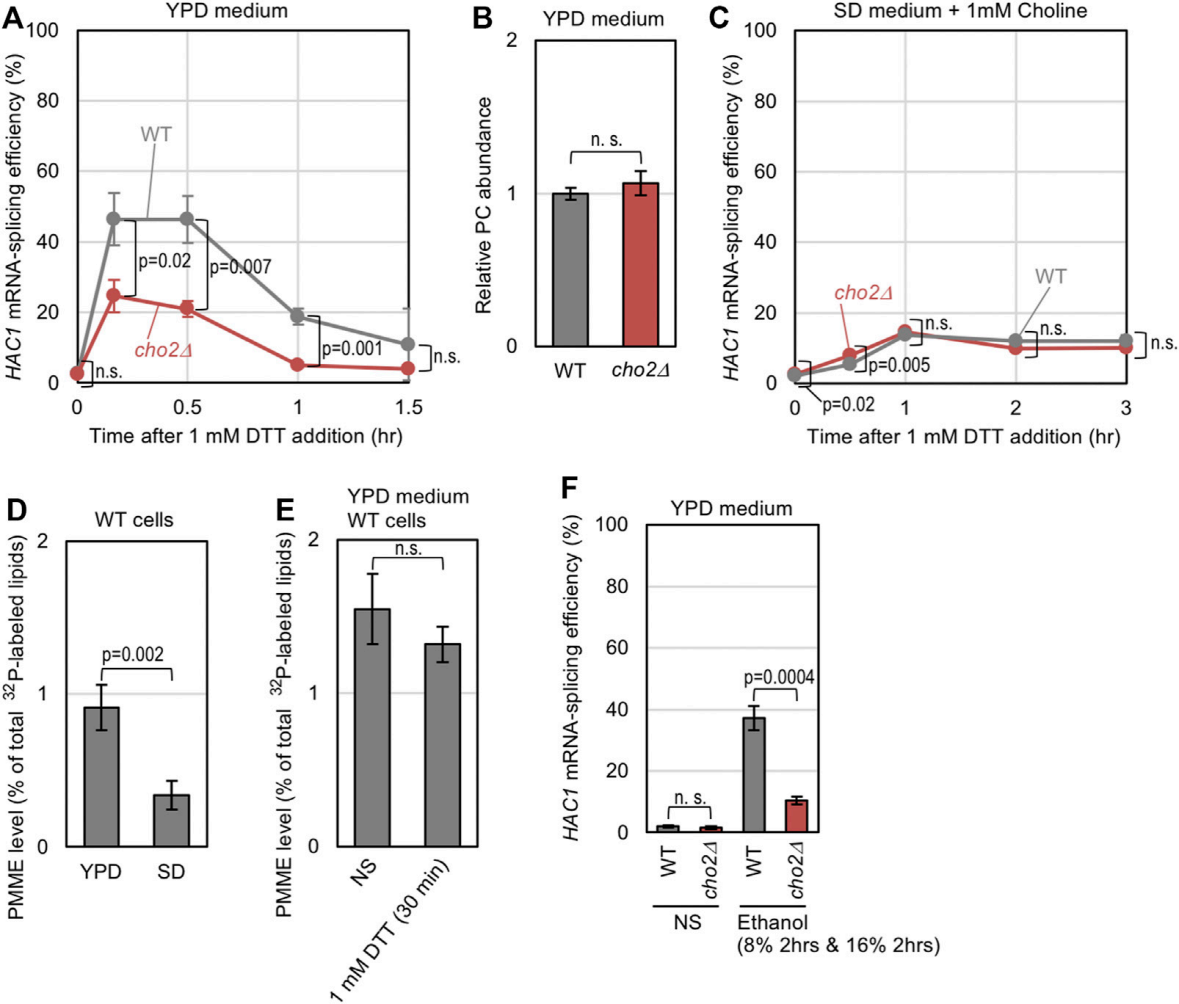
Possible involvement of endogenously accumulated PMME in the UPR level of yeast cells. **(A)** Wild-type (WT) yeast strain BY4741 and its *cho2Δ* mutant were grown in YPD medium, into which 1 mM DTT was added before further culturing for the indicated durations. The cells were then checked for *HAC1*-mRNA splicing. **(B)** BY4741 (WT) and its *cho2Δ* mutant were grown in YPD medium. After harvesting, their lipidic extracts were assayed for PC abundance as described in the *Materials and Methods* section. **(C)** The same experiment as shown in **(A)** was performed, except that SD medium containing 1 mM choline was used instead of YPD medium. **(D)** BY4741 cells (WT cells) were grown in YPD or SD medium containing ^32^P-orthophosphate. After harvesting, their lipidic extracts were assayed for the PMME levels as shown in Supplementary Figure S1. **(E)** BY4741 cells (WT cells) were grown in YPD medium containing ^32^P-orthophosphate, into which 1 mM DTT was added (or not added (non-stress: NS)) before further culturing for 30 min. After harvesting, their lipidic extracts were assayed for PMME levels as shown in Supplementary Figure S1. **(F)** BY4741 (WT) and its *cho2Δ* mutant were grown in YPD medium, and were stressed by a two-step addition of ethanol into the medium (culturing with 8% ethanol for 2 h followed by a 2-h culture with a higher concentration (16%) of ethanol) or remained non-stressed (NS). The cells were then checked for *HAC1*-mRNA splicing. ns, not significant (*p* > 0.05%).

Lastly, we investigated whether the endogenous level of PMME affects ER-stress status in yeast cells. A low dose of DTT (1 mM) induced *HAC1*-mRNA splicing in wild-type cells more strongly than in *cho2Δ* cells when they were cultured in standard nutrient-rich YPD medium (Figure 5A). As YPD medium contains free choline, the PC abundance did not differ between wild-type and *cho2Δ* cells (Figure 5B). Thus, we assume that wild-type cells are prone to ER stress owing to endogenously accumulated PMME. Cells were then cultured in SD medium containing choline, which was added to support the growth of *cho2Δ* cells, and were stressed by 1mM DTT. Figure 5C indicates that, in this case, wild-type cells and *cho2Δ* cells showed similar *HAC1* mRNA-splicing profiles, suggesting that endogenously accumulated PMME does not potentiate ER stress in cells cultured in SD medium. The different outcomes between YPD and SD media can be explained by the result shown in Figure 5D, which indicates that the PMME abundance was considerably lower in wild-type cells cultured in SD medium than those cultured in YPD medium. On the other hand, it is unlikely that ER stress affected cellular PMME abundance (Figure 5E).

We next examined if PMME aggravates ER stress that is induced by a stress stimulus besides DTT. We previously reported that ethanol stress impairs ER protein folding and activates Ire1 (Miyagawa et al., 2014). As shown in Figure 5F, the *cho2Δ* mutation attenuated the *HAC1*-mRNA splicing induced by ethanol in cells cultured in YPD medium.

## Discussion

As recently reviewed by Fun and Thibault (2020), it is widely accepted that LBS causes ER stress and activates ER-stress sensors, which include Ire1, in a wide variety of eukaryotic species. A prominent example of LBS is the saturation of phospholipid acyl tails (Pineau et al., 2009). Volmer et al. (2013) and Halbleib et al. (2017) proposed that the transmembrane domain of Ire1 directly monitors the characteristics of acyl tails. The aberrant composition of the polar head of phospholipids can also induce ER stress. The result shown in Figure 1A confirms a previous proposal by Thibault et al. (2012), who argued that the UPR is provoked upon PC deficiency in *cho2Δ* or *opi3Δ* yeast cells cultured in choline-free medium. The transmembrane-domain amphipathic helix of Ire1 is likely to contribute, directly or indirectly, to sensing this stress (Ho et al., 2020). However, here we propose that another factor is hidden under this PC-deficiency scenario.

In the presence of extracellularly supplemented choline, only the *opi3Δ* mutation, but not the *cho2Δ* mutation or the *cho2Δ opi3Δ* double mutation, induced the UPR and exhibited a synthetic growth defect with the *ire1Δ* mutation (Figure 1). Ye et al. (2017) proposed that impairment of the PE-to-PC sequential methylation causes accumulation of the methyl donor S-adenosylmethionine, which leads to hypermethylation of histones. However, this scenario is unlikely to account for the UPR evocation shown here, because the UPR in *opi3Δ* cells was compromised by further introduction of the *cho2Δ* mutation (Figure 1C). Hence, we propose that PMME *per se* provokes UPR in yeast cells. Consistent with this idea, Cho2 overexpression induced UPR (Figure 1D). Moreover, in yeast cells, ER stress induced by DTT or tunicamycin was aggravated by the extracellular addition of MME, which increased the cellular PMME level probably via the Kennedy pathway (Figures 2A, B, D, G). We also demonstrated that MME induced ER stress in mammalian cells (Figure 2H). Therefore, disturbance of the PC-biosynthesis pathway causes ER stress via two different ways, namely PC deficiency and PMME accumulation, in yeast cells and possibly in mammalian cells. In other words, PMME has an unfavorable biological propert(ies) that is not possessed by PE and PC.

In this study, we also investigated the ER stress-inducing mechanism of PMME. Shyu et al. (2019) reported that various ER-located transmembrane proteins are quickly degraded in *opi3Δ* cells under choline depletion conditions, although such effect was not observed when choline was extracellularly supplemented. Therefore, this scenario is unlikely to explain the toxicity mechanism of PMME. On the other hand, when highly accumulated in *opi3Δ* cells, PMME is likely to cause LBS, which is sensed by the transmembrane domain of Ire1 (Figure 3A). However, we do not think that Ire1 directly monitors the hydrophilic-head composition of phospholipids. As shown in Figure 3B, the *opi3Δ* mutation increased the proportion of lauric acid (C12:0) in lipid fatty acids. We assume that PMME may inhibit fatty-acid elongation. It is possible that, based on its luminal-domain structure, Ire1 self-associates as a result of unevenness in the lipid-bilayer thickness (Covino et al., 2018). This explains the scenario by which PMME activates Ire1 by changing the hydrophobic-tail composition of phospholipids. On the other hand, a lower-level accumulation of PMME is likely to disturb protein folding in the ER when combined with other ER-stress stimuli (Figures 2, 4). Therefore, we presume that PMME may negatively affect multiple cellular events.

Extracellular supplementation of MME alone did not induce UPR in yeast cells, probably because it increased the cellular PMME level only moderately (Figures 2A, D; Supplementary Figure S3). However, ER stress induced by other ER-stress stimuli was aggravated by MME (Figures 2D, G). Based on our observations shown in Figure 5, we presume that a similar phenomenon occurs in yeast cells cultured in the standard nutrient-rich medium YPD even without extracellular supplementation of MME. For an unknown reason, the cellular level of PMME was higher in YPD-cultured cells than SD-cultured cells. Intriguingly and presumably, this leads to high sensitivity of YPD-cultured cells to the ER-stress stimuli, DTT and ethanol. It should be also noted that, although PMME leads to the activation of Ire1, ER stress is unlikely to change the cellular PMME level (Figure 5E). We argue that ER stress is aggravated under conditions in which cells endogenously and spontaneously carry an relatively high amount of PMME.

Sakakibara et al. (2015) reported that mitophagy is inhibited by PMME accumulation in *opi3Δ* yeast cells. Intriguingly, Atg8 conjugated with PMME was not efficiently delipidated in an *in vitro* experiment performed by Sakakibara et al. (2015). To the best of our knowledge, this is the first report to propose a direct inhibitory effect of PMME on a specific biochemical process. Here, we propose that PMME exerts a biological effect(s) even when it accumulates at an endogenous and spontaneous level in cells.

In conclusion, here we revealed that a low-abundance metabolic intermediate, PMME, is hazardous and causes ER stress. Unlike yeast cells, a sole methyltransferase, namely PE *N*-methyltransferase (PEMT), has been thought to be responsible for the PE-to-PC sequential methylation in hepatocytes (Vance, 2014). However, Sprenger et al. (2021) recently reported that PMME is highly accumulated in PEMT gene knockout mice, suggesting that, as well as in yeast cells, PE-to-PMME methylation and PMME-to-PC methylation are performed by different methyltransferases in mammalian cells. In mammals, PEMT is mainly expressed in the liver, which serves as the predominant site of PE-to-PC sequential methylation (Vance, 2014). PEMT gene knockout induces ER stress in murine hepatocytes, possibly leading to steatohepatitis (Gao et al., 2015). We speculate that, in addition to PC shortage, PMME accumulation may account for this phenomenon. Song et al. (2005) and Dong et al. (2007) reported that a mutation in the PEMT gene is linked to human non-alcoholic fatty liver disease and non-alcoholic steatohepatitis. Therefore, exploring the relationship between PMME and human liver diseases may be noteworthy in future studies.

## Data Availability

The raw data supporting the conclusion of this article will be made available by the authors, without undue reservation.

## References

[B1] Boeke J. D. La Croute F. Fink G. R. (1984). A Positive Selection for Mutants Lacking Orotidine-5′-Phosphate Decarboxylase Activity in Yeast: 5-Fluoro-Orotic Acid Resistance. Mol. Gen. Genet. 197, 345–346. 10.1007/bf00330984 6394957

[B2] Collart M. A. Oliviero S. (2001). Preparation of Yeast RNA. Curr. Protoc. Mol. Biol. Chapter 13, Unit13.12. 10.1002/0471142727.mb1312s23 18265096

[B3] Covino R. Hummer G. Ernst R. (2018). Integrated Functions of Membrane Property Sensors and a Hidden Side of the Unfolded Protein Response. Mol. Cell 71, 458–467. 10.1016/j.molcel.2018.07.019 30075144

[B4] Cox J. S. Shamu C. E. Walter P. (1993). Transcriptional Induction of Genes Encoding Endoplasmic Reticulum Resident Proteins Requires a Transmembrane Protein Kinase. Cell 73, 1197–1206. 10.1016/0092-8674(93)90648-a 8513503

[B5] de Kroon A. I. P. M. (2007). Metabolism of Phosphatidylcholine and its Implications for Lipid Acyl Chain Composition in *Saccharomyces cerevisiae* . Biochim. Biophys. Acta Mol. Cell Biol. Lipids 1771, 343–352. 10.1016/j.bbalip.2006.07.010 17010666

[B6] Dong H. Wang J. Li C. Hirose A. Nozaki Y. Takahashi M. (2007). The Phosphatidylethanolamine N-Methyltransferase Gene V175M Single Nucleotide Polymorphism Confers the Susceptibility to NASH in Japanese Population. J. Hepatol. 46, 915–920. 10.1016/j.jhep.2006.12.012 17391797

[B7] Fun X. H. Thibault G. (2020). Lipid Bilayer Stress and Proteotoxic Stress-Induced Unfolded Protein Response Deploy Divergent Transcriptional and Non-transcriptional Programmes. Biochim. Biophys. Acta Mol. Cell Biol. Lipids 1865, 158449. 10.1016/j.bbalip.2019.04.009 31028913

[B8] Gao X. van der Veen J. N. Vance J. E. Thiesen A. Vance D. E. Jacobs R. L. (2015). Lack of Phosphatidylethanolamine N-Methyltransferase Alters Hepatic Phospholipid Composition and Induces Endoplasmic Reticulum Stress. Biochim. Biophys. Acta Mol. Basis Dis. 1852, 2689–2699. 10.1016/j.bbadis.2015.09.006 26391255

[B9] Gardner B. M. Walter P. (2011). Unfolded Proteins Are Ire1-Activating Ligands That Directly Induce the Unfolded Protein Response. Science 333, 1891–1894. 10.1126/science.1209126 21852455 PMC3202989

[B10] Gibellini F. Smith T. K. (2010). The Kennedy Pathway--*De Novo* Synthesis of Phosphatidylethanolamine and Phosphatidylcholine. IUBMB Life 62, 414–428. 10.1002/iub.337 20503434

[B11] Gueldener U. Heinisch J. Koehler G. J. Voss D. Hegemann J. H. (2002). A Second Set of loxP Marker Cassettes for Cre-Mediated Multiple Gene Knockouts in Budding Yeast. Nucleic Acids Res. 30, e23. 10.1093/nar/30.6.e23 11884642 PMC101367

[B12] Halbleib K. Pesek K. Covino R. Hofbauer H. F. Wunnicke D. Hänelt I. (2017). Activation of the Unfolded Protein Response by Lipid Bilayer Stress. Mol. Cell 67, 673–684. 10.1016/j.molcel.2017.06.012 28689662

[B13] Ho N. Yap W. S. Xu J. Wu H. Koh J. H. Goh W. W. B. (2020). Stress Sensor Ire1 Deploys a Divergent Transcriptional Program in Response to Lipid Bilayer Stress. J. Cell Biol. 219, e201909165. 10.1083/jcb.201909165 32349127 PMC7337508

[B14] Hou N. S. Gutschmidt A. Choi D. Y. Pather K. Shi X. Watts J. L. (2014). Activation of the Endoplasmic Reticulum Unfolded Protein Response by Lipid Disequilibrium without Disturbed Proteostasis *In Vivo* . Proc. Natl. Acad. Sci. 111, E2271–E2280. 10.1073/pnas.1318262111 24843123 PMC4050548

[B15] Kanki T. Furukawa K. Yamashita S.-i. (2015). Mitophagy in Yeast: Molecular Mechanisms and Physiological Role. Biochim. Biophys. Acta Mol. Cell Res. 1853, 2756–2765. 10.1016/j.bbamcr.2015.01.005 25603537

[B16] Kanki T. Wang K. Cao Y. Baba M. Klionsky D. J. (2009). Atg32 is a Mitochondrial Protein that Confers Selectivity During Mitophagy. Dev. Cell 17, 98–109. 10.1016/j.devcel.2009.06.014 19619495 PMC2746076

[B17] Kimata Y. Ishiwata-Kimata Y. Yamada S. Kohno K. (2006). Yeast Unfolded Protein Response Pathway Regulates Expression of Genes for Anti-Oxidative Stress and for Cell Surface Proteins. Genes Cells 11, 59–69. 10.1111/j.1365-2443.2005.00921.x 16371132

[B18] Kimata Y. Ishiwata-Kimata Y. Ito T. Hirata A. Suzuki T. Oikawa D. (2007). Two Regulatory Steps of ER-Stress Sensor Ire1 Involving its Cluster Formation and Interaction with Unfolded Proteins. J. Cell Biol. 179, 75–86. 10.1083/jcb.200704166 17923530 PMC2064738

[B19] Kimata Y. Kohno K. (2011). Endoplasmic Reticulum Stress-Sensing Mechanisms in Yeast and Mammalian Cells. Curr. Opin. Cell Biol. 23, 135–142. 10.1016/j.ceb.2010.10.008 21093243

[B20] Kimata Y. Oikawa D. Shimizu Y. Ishiwata-Kimata Y. Kohno K. (2004). A Role for BiP as an Adjustor for the Endoplasmic Reticulum Stress-Sensing Protein Ire1. J. Cell Biol. 167, 445–456. 10.1083/jcb.200405153 15520230 PMC2172501

[B21] Lagace T. A. Ridgway N. D. (2013). The Role of Phospholipids in the Biological Activity and Structure of the Endoplasmic Reticulum. Biochim. Biophys. Acta Mol. Cell Res. 1833, 2499–2510. 10.1016/j.bbamcr.2013.05.018 23711956

[B22] Le Q. G. Kimata Y. (2021). Multiple Ways for Stress Sensing and Regulation of the Endoplasmic Reticulum-Stress Sensors. Cell Struct. Funct. 46, 37–49. 10.1247/csf.21015 33775971 PMC10511038

[B23] Mai C. T. Le Q. G. Ishiwata-Kimata Y. Takagi H. Kohno K. Kimata Y. (2018). 4-Phenylbutyrate Suppresses the Unfolded Protein Response without Restoring Protein Folding in *Saccharomyces cerevisiae* . FEMS Yeast Res. 18, foy016. 10.1093/femsyr/foy016 29452364

[B24] Mai T. C. Ishiwata-Kimata Y. Le Q. G. Kido H. Kimata Y. (2019). Dispersion of Endoplasmic Reticulum-Associated Compartments by 4-Phenyl Butyric Acid in Yeast Cells. Cell Struct. Funct. 44, 173–182. 10.1247/csf.19023 31619600

[B25] Merksamer P. I. Trusina A. Papa F. R. (2008). Real-time Redox Measurements During Endoplasmic Reticulum Stress Reveal Interlinked Protein Folding Functions. Cell 135, 933–947. 10.1016/j.cell.2008.10.011 19026441 PMC2739138

[B26] Miyagawa K.-I. Ishiwata-Kimata Y. Kohno K. Kimata Y. (2014). Ethanol Stress Impairs Protein Folding in the Endoplasmic Reticulum and Activates Ire1 in *Saccharomyces cerevisiae* . Biosci. Biotechnol. Biochem. 78, 1389–1391. 10.1080/09168451.2014.921561 25130742

[B27] Okamoto K. Kondo-Okamoto N. Ohsumi Y. (2009). Mitochondria-Anchored Receptor Atg32 Mediates Degradation of Mitochondria via Selective Autophagy. Dev. Cell 17, 87–97. 10.1016/j.devcel.2009.06.013 19619494

[B28] Phuong H. T. Ishiwata-Kimata Y. Nishi Y. Oguchi N. Takagi H. Kimata Y. (2021). Aeration Mitigates Endoplasmic Reticulum Stress in *Saccharomyces cerevisiae* Even Without Mitochondrial Respiration. Microb. Cell 8, 77–86. 10.15698/mic2021.04.746 33816593 PMC8010904

[B29] Pineau L. Colas J. Dupont S. Beney L. Fleurat-Lessard P. Berjeaud J.-M. (2009). Lipid-Induced ER Stress: Synergistic Effects of Sterols and Saturated Fatty Acids. Traffic 10, 673–690. 10.1111/j.1600-0854.2009.00903.x 19302420

[B30] Promlek T. Ishiwata-Kimata Y. Shido M. Sakuramoto M. Kohno K. Kimata Y. (2011). Membrane Aberrancy and Unfolded Proteins Activate the Endoplasmic Reticulum Stress Sensor Ire1 in Different Ways. Mol. Biol. Cell 22, 3520–3532. 10.1091/mbc.e11-04-0295 21775630 PMC3172275

[B31] Sakakibara K. Eiyama A. Suzuki S. W. Sakoh‐Nakatogawa M. Okumura N. Tani M. (2015). Phospholipid Methylation Controls Atg32‐Mediated Mitophagy and Atg8 Recycling. EMBO J. 34, 2703–2719. 10.15252/embj.201591440 26438722 PMC4641534

[B32] van der Sanden M. H. M. Houweling M. Golde L. M. G. v. Vaandrager A. B. (2003). Inhibition of Phosphatidylcholine Synthesis Induces Expression of the Endoplasmic Reticulum Stress and Apoptosis-Related Protein CCAAT/Enhancer-Binding Protein-Homologous Protein (CHOP/GADD153). Biochem. J. 369, 643–650. 10.1042/bj20020285 12370080 PMC1223098

[B33] Sasser M. (2001). Identification of Bacteria by Gas Chromatographyof Cellular Fatty Acids. Newark, DE: MIDI Inc.

[B34] Shields D. J. Altarejos J. Y. Wang X. Agellon L. B. Vance D. E. (2003). Molecular Dissection of the S-Adenosylmethionine-Binding Site of Phosphatidylethanolamine N-Methyltransferase. J. Biol. Chem. 278, 35826–35836. 10.1074/jbc.m306308200 12842883

[B35] Shyu P. Ng B. S. H. Ho N. Chaw R. Seah Y. L. Marvalim C. (2019). Membrane Phospholipid Alteration Causes Chronic ER Stress through Early Degradation of Homeostatic ER-Resident Proteins. Sci. Rep. 9, 8637. 10.1038/s41598-019-45020-6 31201345 PMC6572771

[B36] Sikorski R. S. Hieter P. (1989). A System of Shuttle Vectors and Yeast Host Strains Designed for Efficient Manipulation of DNA in *Saccharomyces cerevisiae* . Genetics 122, 19–27. 10.1093/genetics/122.1.19 2659436 PMC1203683

[B37] Song J. da Costa K. A. Fischer L. M. Kohlmeier M. Kwock L. Wang S. (2005). Polymorphism of thePEMTgene and Susceptibility to Nonalcoholic Fatty Liver Disease (NAFLD). FASEB J. 19, 1266–1271. 10.1096/fj.04-3580com 16051693 PMC1256033

[B38] Sprenger R. R. Hermansson M. Neess D. Becciolini L. S. Sørensen S. B. Fagerberg R. (2021). Lipid Molecular Timeline Profiling Reveals Diurnal Crosstalk Between the Liver and Circulation. Cell Rep. 34, 108710. 10.1016/j.celrep.2021.108710 33535053

[B39] Thibault G. Shui G. Kim W. McAlister G. C. Ismail N. Gygi S. P. (2012). The Membrane Stress Response Buffers Lethal Effects of Lipid Disequilibrium by Reprogramming the Protein Homeostasis Network. Mol. Cell 48, 16–27. 10.1016/j.molcel.2012.08.016 23000174 PMC3496426

[B40] Tran D. M. Takagi H. Kimata Y. (2019). Categorization of Endoplasmic Reticulum Stress as Accumulation of Unfolded Proteins or Membrane Lipid Aberrancy Using Yeast Ire1 Mutants. Biosci. Biotechnol. Biochem. 83, 326–329. 10.1080/09168451.2018.1530098 30319071

[B41] Travers K. J. Patil C. K. Wodicka L. Lockhart D. J. Weissman J. S. Walter P. (2000). Functional and Genomic Analyses Reveal an Essential Coordination Between the Unfolded Protein Response and ER-Associated Degradation. Cell 101, 249–258. 10.1016/s0092-8674(00)80835-1 10847680

[B42] Tsuchiya Y. Saito M. Kadokura H. Miyazaki J.-i. Tashiro F. Imagawa Y. (2018). IRE1-XBP1 Pathway Regulates Oxidative Proinsulin Folding in Pancreatic β Cells. J. Cell Biol. 217, 1287–1301. 10.1083/jcb.201707143 29507125 PMC5881499

[B43] Vance D. E. (2014). Phospholipid Methylation in Mammals: From Biochemistry to Physiological Function. Biochim. Biophys. Acta 1838, 1477–1487. 10.1016/j.bbamem.2013.10.018 24184426

[B44] Volmer R. van der Ploeg K. Ron D. (2013). Membrane Lipid Saturation Activates Endoplasmic Reticulum Unfolded Protein Response Transducers through Their Transmembrane Domains. Proc. Natl. Acad. Sci. 110, 4628–4633. 10.1073/pnas.1217611110 23487760 PMC3606975

[B45] Walter P. Ron D. (2011). The Unfolded Protein Response: From Stress Pathway to Homeostatic Regulation. Science 334, 1081–1086. 10.1126/science.1209038 22116877

[B46] Ye C. Sutter B. M. Wang Y. Kuang Z. Tu B. P. (2017). A Metabolic Function for Phospholipid and Histone Methylation. Mol. Cell 66, 180–193. 10.1016/j.molcel.2017.02.026 28366644 PMC5482412

