# Supplementary material for: Induction and Aggravation of the Endoplasmic-Reticulum Stress by Membrane-Lipid Metabolic Intermediate Phosphatidyl-N-Monomethylethanolamine
Source: Front Cell Dev Biol. 2022 Jan 6;9:743018. doi: 10.3389/fcell.2021.743018 (PMC8770322; doi:10.3389/fcell.2021.743018)
Supplement: Supplementary file 1 [file DataSheet1.PDF]

Supplemental Figure S1

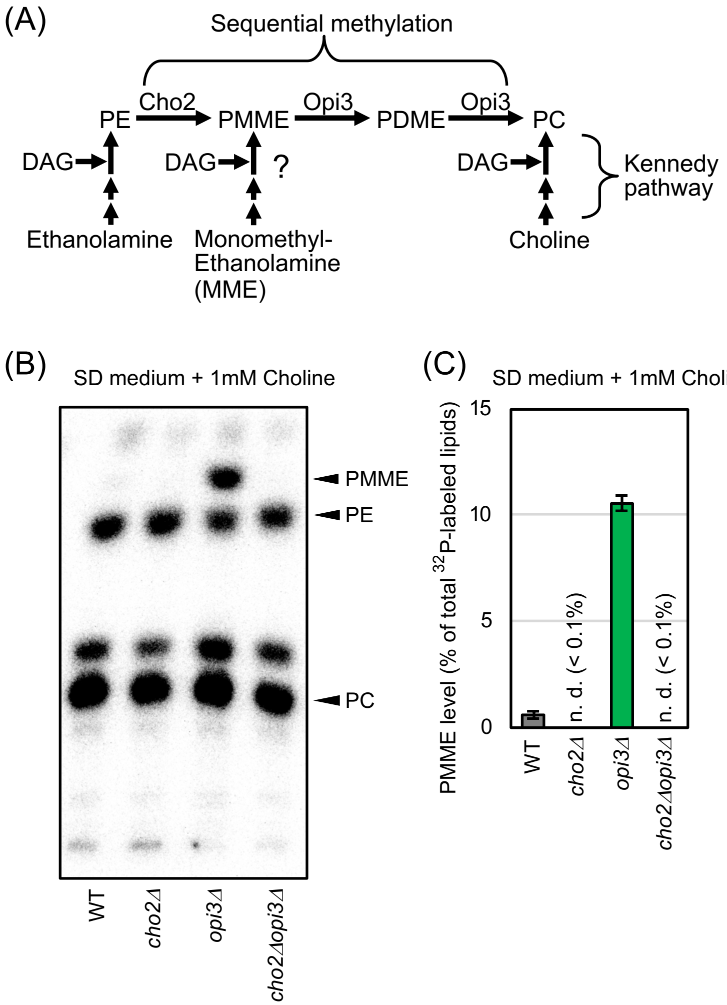

Figure S1 PMME was accumulated in *opi3Δ* yeast cells.
(A) Biosynthetic pathways of PC in yeast cells. DAG: diacylglycerol; PC: phosphatidylcholine; PDME: phosphatidyldimethylethanolamine; PE: hosphatidylethanolamine; PMME: phosphatidylmonomethylethanolamine. (B) Wild-type (WT) yeast strain BY4741 and its gene-deletion mutants were grown in SD medium containing ^{32}P-orthophosphate and 1-mM choline, and their lipidic extracts were run on a TLC plate, which was then subjected to autoradiography. (C) The autoradiogram shown in panel B was analyzed for calculation of the ratios of the PMME signal strength against the total signal strength.

Supplemental Figure S2

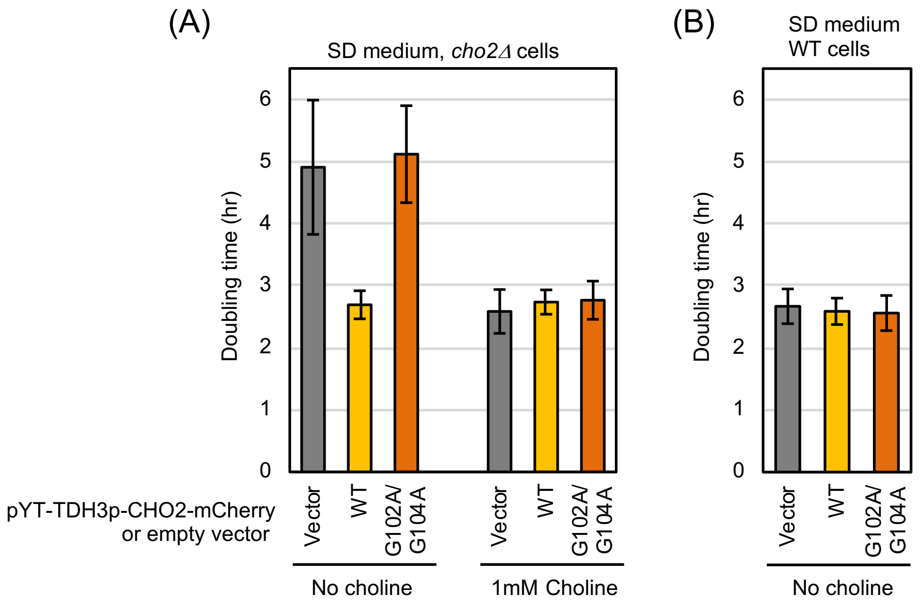

Figure S2 Effect of *TDH3* promoter-controlled expression of Cho2-mCherry on growth of yeast cells.
BY4741 (WT cells) or its *cho2Δ* derivative transformed with the Cho2-mCherry expression plasmid pYT-TDH3p-CHO2-mCherry (wild-type; WT), its G102A/G104A Cho2-mCherry variant, or the control empty vector pRS316 were checked for their growth rates in SD medium supplemented or not with 1-mM choline.

Supplemental Figure S3

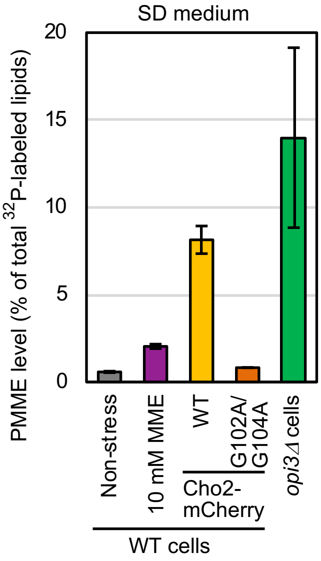

Figure S3 Cellular PMME level under various conditions.
BY4741 cells (WT cells) or *its opi3Δ* mutant were grown in SD medium containing ^{32}P-orthophosphate, and were checked for the cellular PMME levels, as done in figure S1. When indicated, cells were treated with 1-mM MME for 1 hr, or contained the Cho2-mCherry expression plasmid pYT-TDH3p-CHO2-mCherry (WT) or its G102A/G104A mutant. *opi3Δ* cells were grown in the presence of 1 mM choline.

Supplemental Figure S4

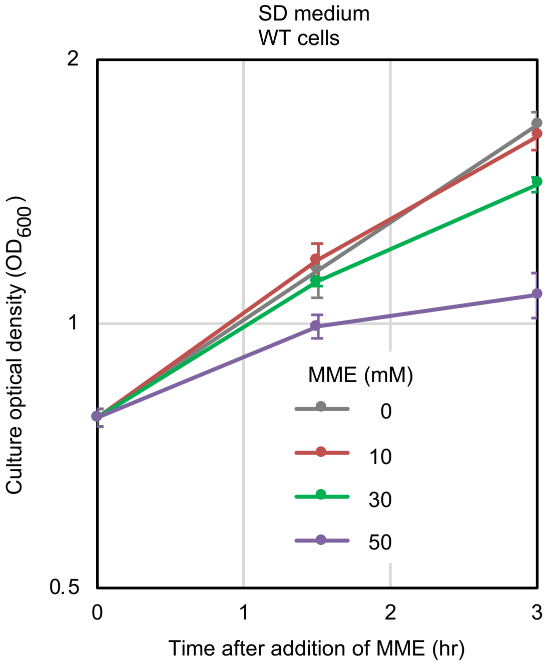

Figure S4 Effect of extracellularly added MME on growth of yeast cells
BY4741 cells (WT cells) were exponentially grown in SD medium. At time 0, MME was added at the indicated concentrations into the cultures, the optical density of which was then monitored.

Supplemental Figure S5

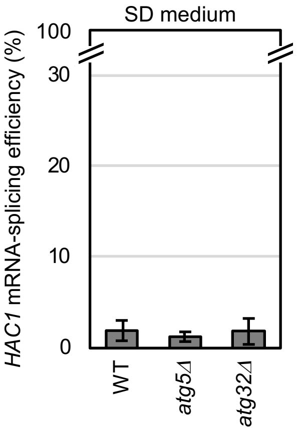

Figure S5 The *atg5Δ* and *atg32Δ* mutations did not induce the UPR in yeast cells.

Wild-type (WT) yeast strain BY4741 and its *atg5Δ* or *atg32Δ* mutant were cultured in SD medium and were checked for the *HAC1*-mRNA splicing.
